# Early kidney damage induced by subchronic exposure to PM_2.5_ in rats

**DOI:** 10.1186/s12989-016-0179-8

**Published:** 2016-12-12

**Authors:** O. G. Aztatzi-Aguilar, M. Uribe-Ramírez, J. Narváez-Morales, A. De Vizcaya-Ruiz, O. Barbier

**Affiliations:** Departamento de Toxicología, Centro de Investigaciones y de Estudios Avanzados del Instituto Politécnico Nacional, Avenida Instituto Politécnico Nacional, No. 2508, Col San Pedro Zacatenco, Ciudad de Mexico, C.P. 07360 Mexico

**Keywords:** Kidney biomarkers, Inflammation, Antioxidant response, Angiotensin and bradykinin systems, Cardiovascular diseases

## Abstract

**Background:**

Particulate matter exposure is associated with respiratory and cardiovascular system dysfunction. Recently, we demonstrated that fine particles, also named PM_2.5_, modify the expression of some components of the angiotensin and bradykinin systems, which are involved in lung, cardiac and renal regulation. The endocrine kidney function is associated with the regulation of angiotensin and bradykinin, and it can suffer damage even as a consequence of minor alterations of these systems. We hypothesized that exposure to PM_2.5_ can contribute to early kidney damage as a consequence of an angiotensin/bradykinin system imbalance, oxidative stress and/or inflammation.

**Results:**

After acute and subchronic exposure to PM_2.5_, lung damage was confirmed by increased bronchoalveolar lavage fluid (BALF) differential cell counts and a decrease of surfactant protein-A levels. We observed a statistically significant increment in median blood pressure, urine volume and water consumption after PM_2.5_ exposure. Moreover, increases in the levels of early kidney damage markers were observed after subchronic PM_2.5_ exposure: the most sensitive markers, β-2-microglobulin and cystatin-C, increased during the first, second, sixth and eighth weeks of exposure. In addition, a reduction in the levels of specific cytokines (IL-1β, IL-6, TNF-α, IL-4, IL-10, INF-γ, IL-17a, MIP-2 and RANTES), and up-regulated angiotensin and bradykinin system markers and indicators of a depleted antioxidant response, were also observed. All of these effects are in concurrence with the presence of renal histological lesions and an early pro-fibrotic state.

**Conclusion:**

Subchronic exposure to PM_2.5_ induced an early kidney damage response that involved the angiotensin/bradykinin systems as well as antioxidant and immune imbalance. Our study demonstrates that PM_2.5_ can induce a systemic imbalance that not only affects the cardiovascular system, but also affects the kidney, which may also overall contribute to PM-related diseases.

**Electronic supplementary material:**

The online version of this article (doi:10.1186/s12989-016-0179-8) contains supplementary material, which is available to authorized users.

## Background

Substantial epidemiological evidence obtained through multi-city and meta-analysis studies has indicated that medium and long-term exposure to particulate matter of less than 2.5 μm (PM_2.5_) is associated with an increase in the incidence of adverse respiratory and cardiovascular events [1].

The health effects reported as a consequence of PM_2.5_ exposure are associated with cellular and molecular inflammation and oxidative stress responses, which are considered to be the underlying mechanisms that drive the cardiopulmonary effects [2–5]. We recently demonstrated that subchronic exposure to coarse, fine and ultrafine particles increases the expression of angiotensin receptor type-1 (AT_1_R) in the lungs and heart. Other genes of the angiotensin and bradykinin endocrine systems, RAS (renin angiotensin system) and KKS (kalikrein kinin system), which are known to be regulated by the kidney, were also up-regulated [6].

The kidneys regulate blood pressure, fluid and sodium homeostasis. These organs are controlled by the sympathetic nervous system [7]. However, renal dysfunction and the development of cardiovascular diseases (CVD) are closely associated. The prevalence of CVD, such as congestive heart failure, coronary artery disease, peripheral vascular disease, and myocardial infarction, amongst others, has been reported in conditions of renal insufficiency and in patients undergoing dialysis, which indicates that there is cross-talk between the kidney and the cardiovascular system [8, 9]. In contrast, the contribution of the CVD to renal dysfunction is poorly understood and has not been adequately studied at a cellular and molecular levels, although there is evidence that diseases such as atherosclerosis [10] and hypertension [11] can contribute to the development of renal dysfunction. The relationship between CVD and renal dysfunction could be considered to be bidirectional given that both factors are independently associated as prognostic indicators. Amann et al. postulated that cardiovascular dysfunction and renal diseases share, as a potential pathogenic mechanism, impaired endothelial function [9]. However, the most important causes of mortality in end-stage kidney disease are CVD and infections, where the infections are thought to be associated with disorders of the innate and adaptive immune responses [12].

Currently, the use of new early molecular biomarkers to establish kidney dysfunction has improved the prognosis for kidney diseases, including acute renal failure [13]. These new markers are proteins that are present in the serum, pass through the glomeruli and can be reabsorbed by the proximal tubules. These proteins include albumin, α-1-glycoprotein (AGP), cystatin-C (Cys-C) and β-2-microglobulin (β2M). Other markers that can be over-expressed after damage to tubular cells include neutrophil gelatinase-associated lipocain (NGAL) and kidney-injury-molecule type-1 (KIM-1). In addition, the presence of these proteins in urine provides a new tool to determine the nephrotoxicity of toxicants such as drugs and inorganic elements as cadmium [14–17].

Few epidemiological approaches have been reported associating PM exposure with a decline of renal function. Estimated glomerular filtration rate (eGFR) reduction has been reported in a one-year study in elderly men from Boston, Massachusetts [75], exposed to PM in a cross sectional study of residents living near a major roadway [18].

There are some experimental studies on rodents that suggest that kidney could be a toxicological target of PM. Nemmar et al., (2009) in a Wistar rat model of acute renal failure, induced by the nephrotoxic cisplatin drug, observed that the intratracheal exposure to diesel exhaust particulates (DEP) enhanced the cisplatin induced kidney damage manifested in serum urea and creatinine, the augment of N-acetyl-β-D-glucosaminidase (NAG) activity, and the depletion of GSH content. Also in this study a decrease in blood of the PO_2_, saturation of O_2_ and changes in hematologic parameters were observed. These results suggest that exposure to particles aggravates renal, pulmonary and systemic effects of cisplatin toxicity, DEP alone did not induced kidney damage [19].

Moreover, Yan et al., (2014) in a diabetic type-1 rat model induced with streptozotocin drug after subchronic exposure to 16 weeks to PM_2.5_, observed an increment in the glycated hemoglobin A1c, IL-6 and fibrinogen, without changes in blood parameters associated with kidney function such as creatinine, microalbumin, NAG, β2M, and blood ureic nitrogen (BUN). Histological analysis showed that PM_2.5_ exposure increased myocarditis, aortic medial thickness, advanced glomerulosclerosis, and a punctual tubular damage of kidney in the diabetic rat model [20].

Clearly, PM_2.5_ exposure exacerbates the complication on diagnosed or induced diseases such as renal failure and diabetes, for that reason pre-existing illnesses and elderly individuals are vulnerable groups to the toxic effects of PM_2.5_.

For these reasons, we hypothesized that exposure to PM triggers an initial endocrine response in the lungs that may affect the heart, and consequently other organs. Cardiovascular effects have been partially attributed to the soluble fraction of particles, in addition cytokines and oxidative stress metabolites, and translocation of the smallest particles also contribute to heart and endothelial damage [2]. On this basis, it is possible to suggest that cytokines and oxidative stress metabolites that circulate throughout the blood stream can reach other organs, such as the kidneys. Moreover, alterations in the vascular tone and a subsequent endothelial dysfunction are factors that contribute to the deterioration of kidney function, and have also been related to PM exposure [3, 21–24]. More knowledge on the possible impact of PM exposure on the renal function is needed.

The goals of the present study were to evaluate the blood pressure status after continuous exposure to PM_2.5_ for a period of eight weeks and to assess renal function using serum creatinine levels and conventional urine testing as well as measuring early kidney damage biomarkers in the urine. In addition, we determined the effects of exposure to PM_2.5_ on the immune and antioxidant responses as well as the effects on the endocrine system by examining the expression of components of the angiotensin and bradykinin systems. Finally, we performed a histological evaluation of the kidney tissue at the end of exposure to demonstrate that the subchronic exposure to PM_2.5_ contributes to the renal response during the pulmonary and cardiovascular toxicity of PM_2.5_.

## Methods

### Animal maintenance

Sprague–Dawley male rats were purchased from Harlan México Laboratories (Mexico city, Mexico). The animals were maintained in a freestanding clean room with a changing station docking port (bioBubble®, Colorado, USA). The rats were provided with filtered water and food *ad libitum* and maintained in a light:dark photoperiod of 12:12 h. All the animals were acclimated and trained for approximately two months for metabolic cage allocation and blood pressure measurements in a rat-holder for blood pressure measurement recording. The procedures were performed in a controlled environment within a bioBubble® station.

### Particle exposure

The particle exposures were performed in whole body chambers associated with a particle concentrator located at CINVESTAV. The particulate concentrator had a particle cutoff size of 2.5 μm. The acute exposure consisted in 3 days, 5 h per day. The subchronic exposure consisted of repeated periods of inhalation for five hours per day, four days per week for eight consecutive weeks. The schedule of exposure was in the morning (8:00 to 13:00 h) from Monday to Thursday in the rainy season (June-August) of 2013.

To evaluate enrichment by the particle concentrator and estimate the particulate chamber concentration, we performed PM_2.5_ ambient air monitoring using a 47-mm teflon filter in a MiniVol samplers with an 5 L∙min^−1^ air flow: the air ambient monitoring period followed the same schedule as the animal exposures. The particulate concentration in the chambers was estimated using a 47-mm teflon filter allocated in a holder inlet which received a 2.5 L min^−1^ constant airflow, which was the same air flow supplied in each chamber. Body mass was considered among control and PM_2.5_ group as air volume displacement, it was adjusted to 1.1 ± 0.2 kg per chamber at the beginning of each weekly experiment, each chamber had a volume of 18 L. The concentrator system was allocated within an enclosed laboratory with controlled air atmosphere (air conditioning and ventilation system), thus temperature and humidity were constant in the exposure chambers. Filters from each week were used for gravimetric analysis. To calculate the particulate enrichment factor, all data were adjusted for the air flow and the ratio between ambient air and the chamber particulate concentrations from each week.

In parallel with the ambient air monitoring, and according to Alfaro-Moreno et al. [25] we collected PM_2.5_, at the same schedule of animal exposure, in cellulose- nitrate membranes with home-made modifications using HiVol samplers, in order to obtain large quantities of PM_2.5_ to determine endotoxin content and reactive oxidant activity using the DTT assay in the PM_2.5_ samples.

We housed the animals three per chamber with four chambers per group for a total of twelve animals each for the filtered air (FA) control group and the PM_2.5_ test group. The animals were randomly selected according to the analyses that were to be performed. For histology analysis, we selected four animal per group; for blood pressure and biochemical analysis, we selected eight per group. From the latter group, we randomly selected six animals for the metabolic cage evaluation to obtain urine and water consumption data.

Twenty-four hours after the last exposure, the animals were anesthetized with 20 mg/kg of sodium pentobarbital and sacrificed by abdominal aorta puncture (terminal exsanguination). Serum samples were obtained to determinate the creatinine concentration using a commercial kit (RANDOX laboratories Ltd, Ardmore, Diamond Road, UK). The kidneys were removed and stored at −70 °C until used.

### Bronchoalveolar lavage fluid (BALF)

In an anaesthetized animal, a transversal incision between the beginning of the rib cage and the head was performed; afterwards the muscle was removed to expose the trachea. Then a cannula was allocated into the trachea until the carina trachea, and fixed to perform a lavage (3×) with a syringe with an isotonic saline solution (at 37 °C) in a 1:15 (volume:body weight) relation. The recovered solution was centrifuged at 2,000 rpm for 5 min, and the cell pellet was suspended in a final volume of 0.5 ml to perform cell counting with trypan blue solution (0.4%; Sigma Aldrich, St. Louis, MO. USA).

Cells for differential cell count (0.1 ml cell suspension) were prepared using cytospin slides and centrifuged (600 rpm, 5 min), and stained with Wright’s stain protocol. One hundred cells per slide (two slides by sample) were scored and a double blind determination was performed. Differential counting was adjusted by the total cell count.

### Dithiothreitol assay and endotoxin levels

To demonstrate the reactive oxidative ability of PM_2.5_, we performed the “dithiothreitol (DTT) assay” as described in De Vizcaya-Ruiz et al. (2006), this assay provides the intrinsic oxidative activity of particulates integrates organic and inorganic components and their redox capability. We combined 10 μg of scraped PM_2.5_ from each week with DTT (Sigma Aldrich, St. Louis, MO. USA), followed by the addition of a DTNB Sigma Aldrich, St. Louis, MO. USA) solution with which the remaining thiol was allowed to react to generate 5-mercapto-2-nitrobenzoic acid, and the absorption at 412 nm was measured. Briefly, the PM_2.5_ samples were incubated at 37 °C with 0.5 M PBS, pH 7.4, in double deionized water with 1 mM DTT for 0 –45 min. The incubation mixture was then mixed with 10% trichloro-acetic acid to stop the reaction; a portion of the mixture was then dissolved in a solution of Tris buffer at pH 8.9 containing 20 mM EDTA and 10 mM DTNB. As an internal control, we used the standard reference material NIST-1649a (U.S. Department of Commerce, Washington. D.C., USA). The redox activity was expressed as the difference between the rate of DTT (nmol) consumed per minute per microgram of sample and the activity observed in the absence of PM.

The endotoxin levels were determined with the Limulus Amebocyte Lysate Pyrochrome Chromogenic Test Kit (Pyrochrome Associates of Cape Cod Incorporated, Falmouth, MA, USA) as recommended by the commercial manufacturer. We used lyophilized endotoxin (*Escherichia coli*; Control Standard Endotoxin, O113:H10). We used 25 μg of scraped PM_2.5_, and the assay was performed in triplicate for each sample.

### Blood pressure measurement

To record the blood pressure, animals were warmed (29 ± 1 °C) for a period of 5–10 min prior each measurement to ensure adequate vasodilatation. Afterwards, the animals were placed in restraints and five blood pressure measurements using a cutoff ring and a transducer were performed (PanLab Harvard Apparatus, Letica 5002. Cornellà de Llobregat, Barcelona, Spain). Acute exposure blood pressure was evaluated at the last day of exposure. The blood pressure in the subchronic exposure was evaluated one day before the initiation of the 8-week exposure (basal measurement) and on the fourth day after every weekly exposure (post-exposure), with an intermediate resting period of two hours for the animals to eat and hydrate to minimize the effects of stress.

PanLab Harvard Apparatus calculated the mean blood pressure (MBP) as follow:

$$ \mathrm{M}\mathrm{B}\mathrm{P} = \mathrm{diastolic}\ \mathrm{pressure} + 0.33\ \left(\mathrm{systolic}\ \mathrm{pressure}\ \hbox{--}\ \mathrm{diastolic}\ \mathrm{pressure}\right). $$

### Metabolic cage

During twelve-hour period, rats were placed in the metabolic cages (Harvard Apparatus, Hollistone, Massachusetts, USA.), which were used to harvest urine, estimate the water intake, and calculate the urinary flow. These data were adjusted for body weight. Food consumption measurements were not performed to avoid contamination of the urine. The samples were centrifuged at 1,000 rpm for 10 min at 4 °C and stored at −70 °C until used. Aliquots of the urine samples from the 8th week were sent to a veterinary laboratory without previous centrifugation (DIVET S.A. de C.V.) for general urine examination (GUE). Serum and urine creatinine were used to estimate glomerular filtration rate (eGFR), which was calculated using the following equation:

$$ eGFR=\left( Urine\; filow\; rate\;\upmu l/ \min /100\;g\; of\; body\; weight\right)\;*\;\Big[ Urine\; Creatinine\;\left( mg/ ml\right)/ Serum\; Creatinine\;\left( mg/ ml\right) $$

### Early kidney damage biomarkers and cytokines determination by Luminex technology

The early kidney damage biomarkers and cytokines were determinate in rat urine and total protein homogenates of the kidney cortex, respectively, using Milliplex magnetic bead-based multi-analyte profiling via Luminex technology, which is a high throughput immunoassay.

We used the rat kidney toxicity magnetic bead panel 2 (RKTX2MAG-37 K, from EMD Millipore, Darmstadt, Germany) to evaluate albumin, α-1 acid glycoprotein (AGP), β-2-microglobulin (β2M), cystatin-C (Cys-C), epidermal growth factor (EGF) and neutrophil gelatinase associated with lipocalin (NGAL). The determination of the early kidney damage biomarkers was performed in duplicate on the urine samples from the first, second, fourth, sixth and eighth weeks. Biomarker analysis was carried out using a Magpix® System (EDM-Millipore, Darmstadt, Germany). The data obtained were adjusted for the urinary volume.

Furthermore, cytokine concentration of IL-1β, IL-6 and TNFα were determined in urine of the PM_2.5_ subchronic exposure using the rat cytokine/chemokine magnetic bead panel (RECYTMAG-65 K, three-plex from EDM-Millipore®, Darmstadt, Germany). Moreover, the concentration of various cytokines (IL-1β, IL-6, TNF-α, IL-4, IL-10, INF-γ, IL-17a) and chemokines (MIP-2 and RANTES) were determined in kidney cortex protein extracts (see protein extraction in Western blot section), with the rat cytokine/chemokine magnetic bead panel (RECYTMAG-65 K, nine-plex from EDM-Millipore®, Darmstadt, Germany). To perform cytokine analysis, the total protein concentration was quantified using the Bradford assay and diluted to a final protein concentration of 1 μg/μl.

### Western blotting

Total protein from the kidney cortex was obtained by homogenization of the tissue in Nonidet-P40 buffer (150 mM NaCl, 1% NP40, 50 mM Tris–HCl pH 8.0 and protease inhibitors), which is used to study cytosolic, membrane-bound or whole cell protein extracts The homogenates were centrifuged at 10,000 rpm 4 °C, and the supernatant was collected and stored at −70 °C until use. A microplate-based Bradford protein assay was used to determine the protein concentrations. An aliquot of 15 μg of protein was loaded onto SDS-polyacrylamide gels and transferred to a PVDF membrane. Then, the membranes were blocked for 1 h with 5% not-fat milk and incubated overnight with primary antibodies to HO-1 (1:1000, rabbit polyclonal, ADI-SPA-895 from Enzo Life Science, NY, USA) or TGF-β (1:800, rabbit, polyclonal, ab66043, Abcam, Cambridge, UK) or from Santa Cruz Biotechnology (Delaware Ave, Santa Cruz, CA, USA): AT_1_R (1:600, rabbit polyclonal AT_1_R 306 antibody, Sc-579), ACE (1:2000; goat polyclonal ACE N-20 antibody, Sc-12184), B_1_R (1:600, goat polyclonal B_1_R M-19 antibody, Sc-15048), KLK-1 (1:800, goat polyclonal KLK-1 V-14 antibody, Sc-23800), γ-GCSc (1:1000, rabbit polyclonal γ-GCSc H-338 antibody, Sc-22755), SOD-2 (1:2000, goat polyclonal SOD-2 N-20 antibody, Sc-18503), or Nrf-2 (1:800, rabbit polyclonal Nrf-2 C-20, sc-722). The blots were then incubated with HRP-labeled secondary antibodies (Bio-Rad Laboratories, Hercules, CA, USA) for 1 h at a dilution of 1:15000. The immunoreactivity was detected using Luminata Western blotting detection reagent (Millipore). The bands were visualized by exposure to X-ray films and photodocumented with a UVP EC3 imaging system (UVP Inc., USA). We used α-actin (a donation of Dr. Hernández-Hernández, CINVESTAV-IPN) as an internal control to correct for protein loading.

### Real time-polymerase chain-reaction

Total RNA was isolated from kidney cortex using the phenol-chloroform method (TRIzol reagent, Invitrogen™, Life Technologies, Thermo Fisher Scientific, Carlsbad, CA, USA). An aliquot of 10 μg of total RNA was subjected to DNAse treatment (Ambion, Turbo DNAse-freeTM, Life Technologies, Carlsbad, CA, USA). cDNA synthesis was performed with 3 μg of total RNA according to the manufacturer’s instructions (SuperScript II, Invitrogen™, Life Technologies, Thermo Fisher Scientific, Carlsbad, CA, USA).

Real-time PCR was performed with a final concentration of 15 ng of cDNA using SYBR Green (Maxima SYBR Green/ROX qPCR master mix, Thermo Fisher Scientific) in an Applied Biosystem StepOne™ instrument.

Specific oligonucleotides (Table 1) were used to evaluate the mRNA levels for the RAS genes *At1r* and *Ace* and for the KKS genes *B1r* and *Klk*-*1*. To evaluate the antioxidant response, we measured *Hmox1*, *Sod2* and *Nrf2*. Finally, we evaluated the expression of pro-collagen-III (*Col3a1*) as a marker of the fibrotic process. We used 18S as the housekeeping gene. The oligonucleotides were synthesized commercially by Sigma Aldrich or Applied Biosystems. PCR cycling conditions were optimized for each oligonucleotide set as follows: 95 °C for 10 min, followed by 40 cycles at 95 °C for 10 s, and 60 °C for 1 min. The data were analyzed using the 2^-ΔΔCt^ method, the calculation for mRNA expression of each gene was performed by correcting the values using 18 s as the housekeeping gene.

**Table 1:** Oligonucleotides used for PCR amplification

Gen	Oligonucleotides	Genbank ID
*At1r*	Fw 5'-AATATTTGGAAACAGCTTGGT-3' Rv 5'-ATGATGATGCAGGTGACTTTG-3'	[GenBank: NM_030985]
*Ace*	Fw 5'-CCAACAAGACTGCCACCTG-3' Rv 5'-GTACTGGTGACATCGAGGTTG-3'	[GenBank: NM_012544]
*B1r*	Fw 5'-AGCATCTTCCTGGTGGTGG-3' Rv 5'-CCAGCAGACCAGGAAGGAG-3'	[GenBank: NM_030851]
*Klk-1*	Fw 5'-CCCTCACCCTGACTTCAAC-3' Rv 5'-TCACACACTGGAGCTCATC-3'	[GenBank: 001005382]
*Nrf2*	Fw 5'-TGCCTTCCTCTGCTGCCATTAG -3' Rv 5'-ATGCTCGGCTGGGACTTGTG -3'	From Applied Biosystems
*Hmox1*	Fw 5'-AGGGAAGGCTTTAAGCTGGTGATG-3' Rv 5'-CCTGCCAGTGGGGCCCATAC-3'	From Applied Biosystems
*Sod2*	Fw 5'-TCCCTATCTCTGTGGTGGTGATG-3' Rv 5'-TATCCTGGTCATAGCCGAAGTCTC-3'	From Applied Biosystems
*Col3a1*	Fw 5'-AGGGTGATCGTGGTGAAAA-3' Rv 5'-TCCTCGATGTCCTTTGATG-3'	[GenBank: NM_032085]
*18S*	Fw 5'-GCAGCTAGGAATAATGGAATA-3' Rv 5'-GACTTTCGTTCTTGATTAATGA-3'	[GenBank: NR_046237]

The gene name, the sequence of the forward (Fw) and reverse (Rv) oligonucleotides, and the Genbank ID or commercial source are shown

### Histology

To perform the histological analysis, the animals were anesthetized and whole-body perfused through the jugular vein with saline solution and then fixed with 4% buffered paraformaldehyde. The kidneys were excised and embedded in paraffin. Slides of 5-μm sections of the tissues were stained with Hematoxylin/Eosin stain and Masson’s Trichrome stain (*HT15*, Sigma Aldrich, USA).

For histological analyses two slides per animal were analyzed. Tubular lesions were determined by the loss of morphological features (e.g. tubular epithelial height of the tubular cuboidal epithelium).

Tubular height was analyzed; five randomized photographs from the kidney cortex were taken with a light microscopy with a 10× objective for each group (four animals per group). Also, we performed twenty randomized measurements of tubular height for each picture. The median was obtained for each microscopy field and per animal.

Collagen deposit was quantified in five randomized fields per sample. Photographs were taken with a 10× objective and analyzed using Image J software. Median was obtained for each kidney sample and used for statistical analysis.

### Statistics analysis

Statistical analyses were performed using SigmaPlot version 11.0. We performed descriptive statistical analysis and produced box-plots to show the medians and interquartile 25–75 ranges. To compare two groups, we performed the U-Mann Whitney test on the basis of the non-normal data. Repeated measures ANOVA was performed for the early kidney biomarkers analysis, water consumption, and urinary flow rate. A Pearson correlation analysis was performed between PM mass, endotoxin, PM_2.5_ redox activity and urine early kidney damage biomarkers. A *p* value ≤0.05 was considered statistically significant. All comparisons were performed relative to the filtered air control group.

## Results

### Exposure description

**Fig. 1:**
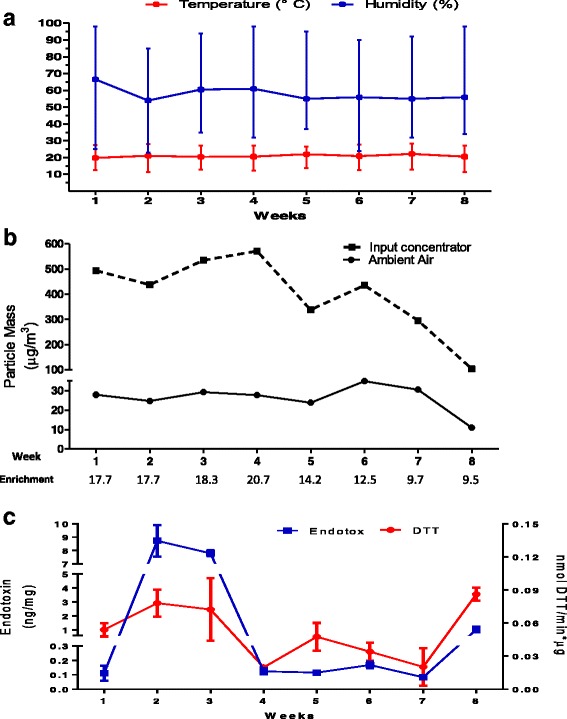
Particulate exposure description. Animal exposure was performed in the raining season in Mexico City. Ambient parameters such as relativity humidity and temperature was monitored by a weather station, it was not observed raining during the schedule exposure. We report the median and the range for each week (**a**). During eight weeks animals exposure (input concentrator) and ambient air were monitored simultaneous, each week was defined as 4 days/week, 5 h/ day. The particulate concentrator enrichment have a minimum enrichment of 9.5 and a maximum of 20.7 times (**b**). Particulate scraped dust from each week was used to determinate the endotoxin content and the oxidative capability of particles by DTT oxidation assay, our data showed that Endotoxin and DTT have the same pattern and the weeks 2, 3 and 8 have the highest values during the exposure (**c**). Each graph point represent the triplicate average ± standar desviation

The animals were exposed in the months of June to August of 2013. These months are considered the rainy season in Mexico City. However, during the acute and subchronic exposure periods, ambient air parameters remained constant: scant rainfall occurred, median percent humidity was 50% with a maximum and minimum percentages of 84 and 32, respectively, and the median temperature was of 21 °C with a range of 11 to 28 °C (Fig. 1a).

During both exposure periods, we determined PM_2.5_ levels in ambient air. Our outdoor air monitoring measurements indicated a median mass concentration of 23.5 μg/m^3^ in the acute exposure period. Moreover, during the subchronic exposure period the median mass concentration was 28 μg/m^3^ (25–30 μg/m^3^), the 25–75 interquartile range is shown in parentheses (Fig. 1b). The gravimetric analysis of the particle concentrator filters indicated a median mass concentration in the exposure chambers for the acute exposure of 445 μg/m^3^, and for the subchronic exposure of 375 μg/m^3^ (300 – 494 μg/m^3^) the 25–75 interquartile range is shown in parentheses. The particulate enrichment for the acute exposure was 19 times and the average for the subchronic exposure was 16 times (9.5 times the lowest and 20.7 times the highest); the lowest enrichment was observed in the last week, in which the lowest ambient air concentration was also recorded (Fig. 1b).

In addition, PM_2.5_ were collected weekly simultaneously with the animal exposures and used to determine the intrinsic particulate oxidative activity with the DTT assay as well as the endotoxin content. We observed an intrinsic oxidative activity of PM_2.5_ in the acute exposure (0.043 nmol DTT/min*μg), however, in the subchronic exposure high oxidative activity on the second, third and the last weeks at approximately 0.08 nmol DTT/min*μg, and the lowest oxidative activities, 0.2 nmol DTT/min*μg, were observed on the fourth and fifth weeks (Fig. 1c). Moreover, we observed in acute exposure an endotoxin concentration of 1 ± 0.08 ng/mg, and in the subchronic exposure observed the highest particulate endotoxin content, up to 8 ng/mg, in the second and third weeks, but during the first, fourth, fifth, sixth and seventh week, the values were approximately 0.1 ng/mg, and during the eighth week, the endotoxin levels were approximately 1 ng/mg (Fig. 1c).

### Lung damage

**Fig. 2:**
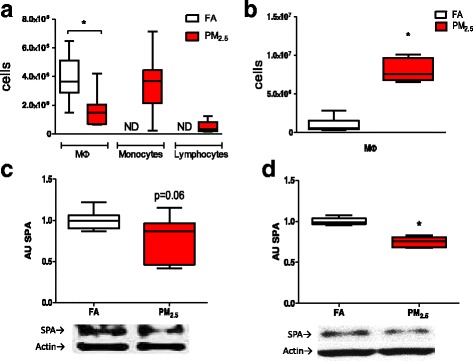
PM_2.5_ exposure induces inflammatory response and a reduction in SPA levels in rat lungs. Rats were acutely (3 days, 5 h/day) and subchronically exposed to concentrated PM_2.5_ (8 weeks, 4 days/week, 5 h/day), and filtered air (FA) as a control group. Subcellular population counts of macrophagues (Mφ), monocytes and lymphocytes from PM_2.5_ bronchialveolar lavage showed an augment after the acute exposure (**a**); however, in subchronic exposure only Mφ count augment in the PM_2.5_ group was observed (**b**). Surfactant protein type-A (SPA) showed a marginal down regulation in the acute exposure to PM_2.5_ (**c**), on the other hand, SPA levels after subchronic exposure to PM_2.5_ decrease statistically (**d**). *in boxplot graphyc indicates statistical significant differences (*p <* 0.05)

To demonstrate that the PM_2.5_ was able to induce a lung response, we evaluated the bronchoalveolar lavage fluid (BALF) cell counts and determined the protein concentration of surfactant protein-A (SPA) as an indicator of molecular damage (Fig. 2). At the end of the subchronic PM_2.5_ exposure, a significant increase in the macrophage (MΦ) counts was observed, and monocytes and lymphocytes were not observed (Fig. 2b). To establish lung inflammation in response to PM_2.5_ differential cell count in lung BALF was performed, we included an independent experiment to establish the acute response of the lung to PM_2.5_ (3 days; 5 h/day exposure). We observed a decrease in the MΦ counts and an increase in monocyte and lymphocyte counts in the BALF of the acutely exposed animals (Fig. 2a).

With respect to the SPA levels, a marginal reduction (*p =* 0.06) was observed in animals acutely exposed to PM_2.5_ (Fig. 2c). This decrease in SPA was statistically significant in the PM_2.5_ eight-week exposure group compared to the FA group (*p <* 0.05; Fig. 2d).

Lung inflammation and an increment in blood pressure was observed after the acute exposure to PM_2.5_, however no effects were observed in kidney parameters: urinary flow, kidney relative weight, plasma creatinine, urine pH, urine specify gravity, and hematuria (Additional file 1: Table S1), thus we excluded the analysis of urinary kidney biomarkers of the acute exposure.

### Blood pressure measurement

In this study, we report the mean blood pressure (MBP) as a physiological parameter of vascular tone on the basis that it is indicative of the perfusion pressure of organs, which could be affected by exposure to PM_2.5_. In subchronic exposure blood pressure was measured before the beginning the exposure and after every weekly exposure. We analyzed MBP using two different approaches: 1) we compared the weekly MBP measurements from the PM_2.5_ group after exposure with the FA group and 2) we compared all the measurements with the basal MBP measurement to evaluate changes in the blood pressure as a result of the experimental exposure procedure (Table 2).

**Table 2:** Mean blood pressure measurements after subchronic exposure to PM_2.5_ and filtered air (FA)

	FA	PM_2.5_
Basal	109.3	109.6
	(103.0–122.3)	(106.6–112.0)
Wk-1	112.3	133.2*^a^
	(107.2–116.7)	(122.7–144.4)
Wk-2	106.9	109.8
	(103.1–115.4)	(102.8–116.7)
Wk-3	122.9	129 ^a=0.06^
	(119.3–132.7)	(113.4–135.7)
Wk-4	116.1	116.9
	(106.7–124.5)	(109.1–122.9)
Wk-5	109.3	131.7*^a^
	(105.0–111.3)	(123.3–136.3)
Wk-6	101.8	108.5
	(97.8–106.6)	(103.2–127.5)
Wk-7	115.5	113.2
	(87.9–131.1)	(104.2–121.7)
Wk-8	107.6	128.1*^a^
	(102.8–114.7)	(125.2–135.6)

The data are shown as the median followed by the 25-75 quartile interval in parentheses.

*Indicates statistically significant differences between the PM_2.5_ and the FA groups for each week (Wk) (*p*<0.05).

^a^Indicates a statistically significant difference from the basal measurement (FA vs FA, and PM_2.5_ vs PM_2.5_).

We observed increases in the MBP after the first, fifth and eighth week of exposure to PM_2.5_ compared to the FA control group. The same differences were observed in the comparison of the PM_2.5_ group measurements to the initial basal measurements in this group. The increased MBP values in the first, fifth and eighth week were driven by increases in the diastolic blood pressure, which showed the same differences as the median (data not shown). Thus, the blood pressure data indicate that exposure to PM_2.5_ can affect the vascular tone and probably the perfusion of organs.

### Hydration state

**Fig. 3:**
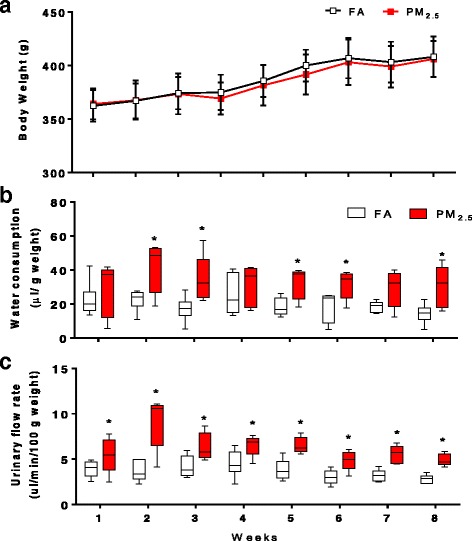
The PM_2.5_ continuous exposure affects the hydric state. Rats were subchronically exposed to concentrated PM_2.5_ (8 weeks, 4 days/week, 5 h/ day). Particulate exposure does not modify the body weight of animals during the eight weeks (**a**). However, the water consumption (**b**) and the urinary flow rate (**c**) increase significantly weekly at the end each exposure week. On boxplot graphyc* indicates statistical differences (*p <* 0.05)

During the eight weeks of exposure, both groups were weighed at the end of every week. We did not observe differences between the body weights of the FA and PM_2.5_ groups (Fig. 3a). In addition, water consumption and the urinary flow rates were measured during the twelve-hour period in the metabolic cages. Subchronic exposure results in a significant increases in the water intake in the second, third, fifth, sixth and eighth weeks (*p <* 0.05; Fig. 3b). On the first, fourth and seventh weeks, the median water consumption in the PM_2.5_ groups was increased, but the difference between the groups was not statistically significant. In addition, the urinary flow rate was increased in the PM_2.5_ group compared to the FA group for all eight weeks (*p <* 0.05; Fig. 3c).

### Urine and kidney function parameters

At the end of the exposure period, no significant differences in the kidney relative weights were observed. However, plasma creatinine increased slightly significantly in the PM_2.5_ group compared to the FA group (*p <* 0.05). The eGRF a decrement in the PM_2.5_ group compared to the FA group (488 vs. 643 μl/min/100 g of body weight). These data suggested kidney damage after subchronic exposure (Table 3).

**Table 3:** Kidney and urine parameters after subchronic of exposure to PM_2.5_

Parameter	FA	PM_2.5_	*P*-value
Kidney relative weight (g/body weight)	0.68 (0.64 –0.72)	0.67 (0.63 –0.71)	0.34
Plasma creatinine (mg/dl)	0.6 (0.5 –0.65)	0.7 (0.65 –0.9)	0.03
eGFR (μl/min/100 g of weight)	569 (541 –828)	483 (386 –548)	0.02
pH	6.8 (6.6 –7)	7 (6.8 –7.3)	0.13
Urine specific gravity	1.02 (1.01 –1.023)	1.013 (1.01 –1.02)	0.09
Hemoglobin	1	2 (1 –4)	0.03
Hematuria^a^	0:6	4:6	0.007

The data are shown as the median followed by the 25–75 quartile interval in parentheses

1 = absent; 2 = limited; 3 = + and 4 = ++

^a^low erythrocytes counts in the urine (frequency is reported)

The urine parameters from the GUE performed on the week 8 from subchronic exposure, such as pH and urine specific gravity, did not show any statistically significant differences. However, a moderate presence of hemoglobin and erythrocytes in the urine from the PM_2.5_ group was observed, whereas these findings were absent in the urine of the FA group. In addition, we observed a statistically significant frequency of high hematuria in the sediment of the urine from the PM_2.5_ group compared to the FA group (Table 3).

### Early kidney damage markers

**Fig. 4:**
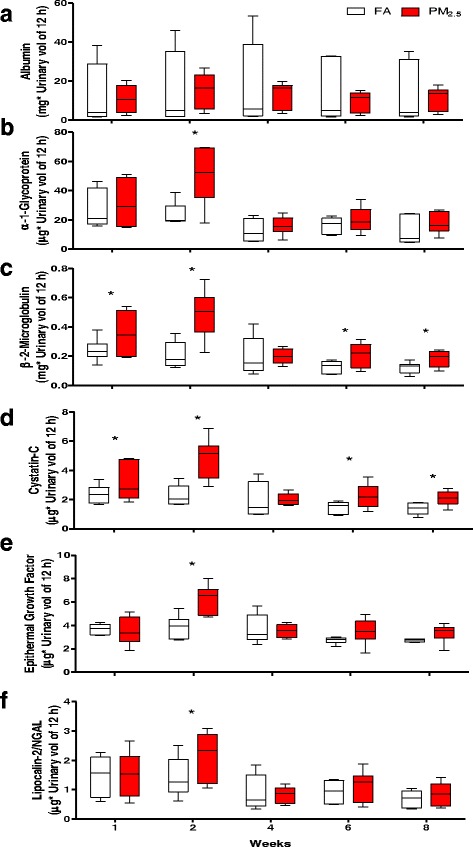
Exposure to PM_2.5_ induces the release of kidney early damage markers in urine. Rats were exposed to concentrated PM_2.5_ (8 weeks, 4 days/week, 5 h/ day). At the end of each week twelve hours-urine was collected by metabolic cage. Six early kidney markers were evaluated by Luminex technology. Albumin does not show statistical differences at any time evaluated (**a**). α-1-glycoprotein (**b**), β-2-Microglobulin (**c**), Cistatin-C (**d**), epithermal growth factor (**e**) and lipocalin-2/NGAL (**f**) have the highest increment in urine levels in the second week. The most sensitive early markers a long to the particle exposure were β-2-Microglobulin and Cistatin-C showed increments on the first, second, sixth, and eighth week. At week 4 any early damage kidney marker showed statistical differences. On boxplot graphyc * indicate statistical differences (*p <* 0.05)

We collected twelve-hour urine from animals after the last day of each exposure, six early kidney damage biomarkers were measured after the first, second, fourth, sixth and eighth weeks of subchronic exposure (Fig. 4). Albumin was detected in urine of both groups after the subchronic exposure, and no statistical difference was observed (Fig. 4a). The AGP (Fig. 4b), EGF (Fig. 4e) and NGAL (Fig. 4f) markers showed statistically significant differences between PM_2.5_ and FA groups only in the second week of exposure. On the other hand, the markers that showed the greatest significant differences were β2M (Fig. 4c) and Cys-C (Fig. 4d), which showed increases in the first, second, sixth and eighth week in the PM_2.5_-exposed groups, with the greatest increase occurring in the second week.

Pearson correlation analysis between endotoxin and intrinsic redox activity of PM_2.5_ and urine early kidney biomarkers was performed (Additional file 1: Table S2), statistical analysis showed a significant correlation of endotoxin with renal biomarkers. DTT assay also showed a positive and significant correlation with early kidney damage biomarkers, although not as strong.

### RAS/KKS endocrine response

**Fig. 5:**
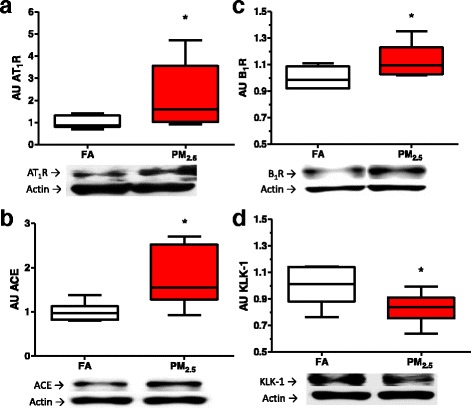
Proteins of angiotensin-, and bradykinin- systems respond at the end of eight weeks to PM_2.5_ exposure. After eight exposure weeks (4 days/week, 5 h/ day) in kidney rats, we observed an augment in angiotensin-converter enzyme (ACE) and angiotensin-receptor type-I (AT_1_R), (**a**) and (**b**) respectively. Bradykinin receptor type-1 (B_1_R) increase and tissue kallikrein (KLK-1) decrease, (**c**) and (**d**) respectively. On boxplot graphyc * indicates statistical differences (*p <* 0.05)

RAS/KKS have been reported to be important regulators of the physiopathology of the kidney and the cardiovascular system. For the RAS, we evaluated the expression of *At1r* and *Ace* in the kidney because these genes are involved in the endocrine pathway and the production of angiotensin, respectively. The mRNA levels of both genes were decreased in the kidney cortex in the group exposed to PM_2.5_ after eight weeks of exposure compared to the FA group (Table 4). However, the protein levels of AT_1_R (Fig. 5a) and ACE (Fig. 5b) were significantly greater in the PM_2.5_ group than in the FA group.

**Table 4:** PM_2.5_ subchronic exposure modulates the mRNA expression of endocrine Angiotensin and Bradykinin system components and the antioxidant response in rat kidney

Gene	FA	PM_2.5_	*p*-value
*At1r*	1 (0.9 –1.16)	0.7 (0.5 –0.87)	0.04
*Ace*	0.9 (0.8 –1.1)	0.5 (0.4 –0.68)	0.002
*B1r*	1 (0.76 –1.52)	4.3 (2.74 –7.17)	0.009
*Klk-1*	1 (0.8 –1.2)	0.5 (0.2 –0.77)	0.004
*Nrf-2*	1 (0.9 –1.1)	1.4 (1.0 –2.5)	0.055
*Hmox1*	1 (0.9 –1.4)	0.7 (0.5 –0.87)	0.008
*Sod2*	0.9 (0.7 –1.2)	0.3 (0.1 –0.4)	0.001

The data are shown as the median followed by the 25–75 quartile interval in parentheses

From the KKS, we evaluated *B1r* and *Klk-1*, two important mediators of vascular tone and the inflammatory response. After eight weeks of exposure to PM_2.5_, we observed a 4-fold increase in the *B1r* mRNA levels compared to the FA group (Table 4), as well as an increase in the protein levels in the PM_2.5_ group (Fig. 5c). On the other hand, the *Klk-1* mRNA levels in the PM_2.5_ group were reduced to half the value of those of the FA group (Table 4), and the same decrease in the KLK-1 protein levels was observed in the PM_2.5_ group compared to the FA group (Fig. 5d).

### Antioxidant response

**Fig. 6:**
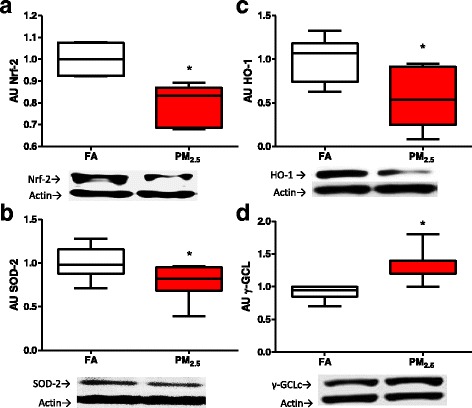
Kidney antioxidants decreased at the end of the eight weeks of PM_2.5_ exposure. After eight exposure weeks (4 days/week, 5 h/ day) in kidney rats we observed a decrement of the antioxidant nuclear transcription factor (Nrf-2) (**a**), and antioxidant enzymes such as mitochondrial superoxide dismutase (SOD-2) and hemoxygenase type-1 (HO-1), (**b**) and (**c**) respectively. However, the gamma-glutamil cysteine ligase heavy chain the catalytic subunit (γ-GCLc) was up-regulated showed the kidney dependence of glutathione resource (**d**) On boxplot graphyc * indicates statistical differences (*p <* 0.05)

The toxicity of PM_2.5_ can be largely explained by the induction of oxidative stress and the inflammatory response. To evaluate the role of oxidative stress, we evaluated the mRNA of *Nrf-2*, *Hmox1* and *Sod2* as molecular elements that respond to oxidative stress. We observed a trend toward an increase in *Nrf-2* mRNA with a marginal significance (*p =* 0.055); however, the mRNA levels of the antioxidant enzymes *Sod2* and *Hmox1* decreased significantly in the PM_2.5_ group compared to the FA group (Table 4). To confirm these results, the protein levels were evaluated. The Nrf-2 protein levels (Fig. 6a) as well as those of SOD-2 (Fig. 6b) and HO-1 (Fig. 6c) were reduced in the kidney cortex of the PM_2.5_ group. In addition, to confirm a possible antioxidant response, we decided to evaluate the protein levels of the catalytic subunit of the heavy chain of gamma-glutamyl cysteine ligase (γ-GCLc), which is involved in the synthesis of glutathione. We observed a modest statistically significant increase in the γ-GCLc levels in the PM_2.5_ group compared to the FA group (Fig. 6d).

### Inflammatory cytokine evaluation

Urine cytokine determination was performed to evaluate pro-inflammatory response (IL-1β, IL-6, and TNFα) of the kidney. We observed that urine cytokine concentrations were below the LUMINEX assay detection limit in PM_2.5_ exposed group. To confirm the inflammatory response to PM_2.5_ subchronic exposure was determined by evaluating a panel of pro- and anti-inflammatory cytokines (IL-6, IL-1β, TNFα, IL-4, IL-10, INF-γ and IL-17a) and chemokines (MIP-2 and RANTES) in kidney cortex homogenates. IL-6 and RANTES were the most abundant cytokines by nanograms per μl concentrations, whereas the remaining cytokines were observed in picograms per μl (Table 5).

**Table 5:** Subchronic exposure to PM_2.5_ decreased the cytokine levels in the total protein of the kidney cortex

Cytokine	FA	PM_2.5_	*P*-value
IL-6 (ng/ul)	10.7 (5.5-11)	1.5 (0.7-4)	0.047
IL-1β (pg/ul)	488.4 (396–531)	297 (242–420)	0.047
TNFα (pg/ul)	47.85 (36–61)	9.9 (6–21)	0.004
IL-4 (pg/ul)	320.7 (228–341)	126 (68–266.7)	0.004
IL-10 (pg/ul)	478.1 (354–537)	199.3 (113–371)	0.03
INF-γ (pg/ul)	371.3 (317–431)	310.6 (192–336)	0.03
IL-17a (pg/ul)	178.6 (93–206)	34 (29–65)	0.004
MIP-2 (pg/ul)	316.2 (200–366)	122.4 (109–161)	0.004
RANTES (ng/ul)	0.98 (0.8–1.14)	1.3 (1.11–1.5)	0.007

The data are shown as the median followed by the 25–75 quartile interval in parentheses

The levels of all of the cytokines in the kidney cortices of the PM_2.5_ group were decreased, with the exception of RANTES, which was slightly increased. All of these differences of the levels in the FA group were statistically significant. Specifically, compared to those of the FA group, the levels of IL-6 in PM_2.5_ kidney cortical homogenates were seven-fold lower; IL-1β was 1.6-fold lower; TNFα was 4.8-fold lower; IL-4 was 2.5-fold lower; IL-10 was 2.4-fold lower; IL-17a was 5.3-fold lower; MIP-2 was 2.6-fold lower; and INF-γ was slightly lower (Table 5).

### Histology and pro-fibrotic state

**Fig. 7:**
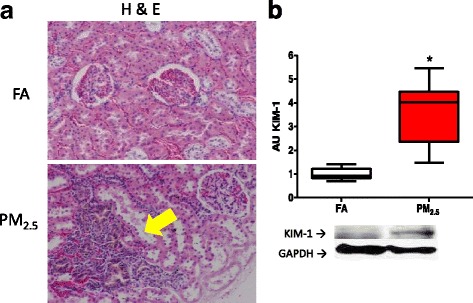
Exposure to PM_2.5_ induces early kidney lesions with the presence of tubular deterioration. After eight exposure weeks (4 days/week, 5 h/ day) in kidney histology we observed tubular lesion of hematoxylin and eosin staining slides (**a**). Tubular damage was corroborate by the immunodetection of Kidney injury molecule-1 (KIM-1) (**b**). On boxplot graphic * indicates statistical differences (*p <* 0.05)

After eight weeks of continuous PM_2.5_ exposure, the histology of the H&E-stained kidney samples demonstrated the presence of tubular lesions with alterations in the tissue structure including a statistical difference (*p <* 0.05) in the reduction in height of the cuboidal epithelium of the tubules; FA 15.4 μm (25–75 quartile 15.2 –16.7) versus PM_2.5_ 9.3 μm (25 –75 quartile 8.1 –10.3) and an intertubular immune cell infiltration (Fig. 7a).

On the basis of the histology, we evaluated the levels of KIM-1 in the total protein extracts of the kidney cortex using Western blotting to confirm the damage in proximal tubules. We observed a statistically significant induction of KIM-1 in the kidney cortex of the PM_2.5_-exposed group; the levels were 4-fold higher than those of the FA group (Fig. 7b).

**Fig. 8:**
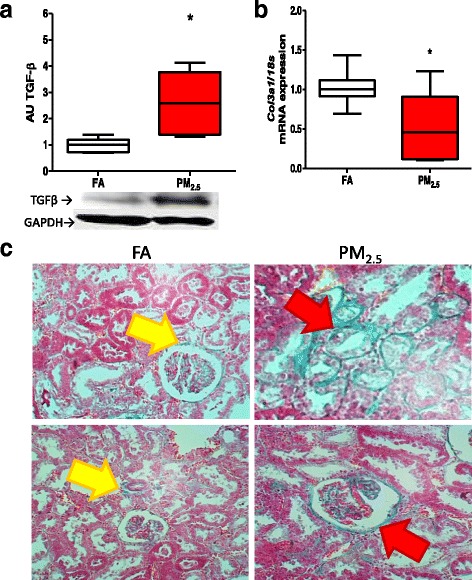
Exposure to PM_2.5_ induces an early pro-fibrosis state. After eight exposure weeks (4 days/week, 5 h/ day) in kidney rat we observed the promotion of fibrosis by the detection of beta- transforming growth factor (TGF-β) (**a**) and the evaluation of pro-collagen-III mRNA (*Col3a1*) by q-PCR, (**b**) Premature deposit of collagen in tubular and glomerulli by the Masson’s trichromic staining (**c**). Yellow and red arrows indicate deposit of colagen in control (FA) and PM_2.5_ groups, respectivetly. On boxplot graphic *indicates statistical differences (*p <* 0.05)

We also evaluated whether PM_2.5_ induced a pro-fibrogenic state at the end of the eight weeks of exposure. TGF-β expression was analyzed as a response to the resolution of inflammation and as an inducer of fibrosis. We observed an approximately three-fold higher level of TGF-β in the total protein extracts of the kidney cortex of the PM_2.5_ group than those of the FA group (Fig. 8a). Finally, we evaluated the expression of *Col3a1* and the presence of collagen in the kidney tissue using Masson’s staining to demonstrate the pro-fibrogenic state. The level of *Col3a1* in the kidney samples from the PM_2.5_ group was lower than that of the FA group (Fig. 8b). However, Masson’s staining indicated the presence of collagen in kidney cortices in the PM_2.5_ group, mainly in the tubular zone and the glomeruli (Fig. 8c). Collagen quantification showed a statistical significant increment in the PM_2.5_ group of 3.2% (1.5 –4.3) in the occupied area by collagen with respect to the FA group 0.55% (0.36 –0.6).

## Discussion

Epidemiological data indicate that PM_2.5_ is one of the main air environmental xenobiotics associated with respiratory and cardiovascular disease mortality and morbidity [1, 5, 26, 27].

The present study provides new evidence that PM_2.5_ exposure can also induce changes in kidney physiology. There are few studies that suggest that PM_2.5_ exposure in human populations or in in vivo rodent models can alter kidney physiological parameters [19, 20]. However, kidney physiology could be compromised as a secondary target organ given the physiological functions of the kidneys, including: 1) blood pressure control; 2) hydration state equilibrium; 3) acid-alkaline homeostasis; 4) endocrine function; and 5) excretion/reabsorption of molecules during urine formation to maintain the body homeostasis.

To investigate whether kidney physiology is affected by PM_2.5_ exposure, we first evaluated the lung damage induced by PM_2.5_ exposure. The data indicated differences between the acute and subchronic inflammatory responses. In the PM_2.5_ acute exposure, cellular recruitment to the BALF was observed, which was characterized by increases in monocytes and lymphocytes accompanied by a reduction in Mφ counts. The latter observation could be explained by the induction of cell death of alveolar Mφ, the presence of PM in high concentrations in the alveolar space and their surface components can activate scavenger receptors from neighboring cells and mediate Mφ apoptosis [28, 29]. On the other hand, during the subchronic exposure, an increase in the number of Mφ in the BALF of the PM_2.5_ exposed group compared with the FA group was observed, suggesting a recovery and activation of the immune response and the recruitment of Mφ by the end of the eight-week exposure. The subchronic exposure to PM_10_ and DEP in rabbits and mice, resulted in an increase in alveolar macrophages was observed as the result of the recruiting of circulating monocytes that differentiated to macrophages [30, 31].

The observed increase in the Mφ cellular population in the BALF after subchronic exposure demonstrated that the repeated PM_2.5_ exposure compromised the lung physiology. This observation was supported by evidence of an alteration in the surfactant system, which is necessary for the regulation of surface tension and contributes to the innate immunological barrier. Surfactant protein-A is a good candidate as a lung damage biomarker because it is down-regulated in lung pathological states, such as idiopathic pulmonary fibrosis [32], adult respiratory distress syndrome, asbestosis and silicosis [33]. In our experimental model, PM_2.5_ exposure decreased lung SPA protein levels after the subchronic exposure. These data suggest that PM_2.5_ can adversely affect pulmonary surface tension and that the antioxidant and innate immune functions of the lung would also be affected because SPA participates in the mechanical functions of the lung, modulates oxidation of the phospholipids by serving as an antioxidant [34], and acts as a type of pulmonary host defense molecule, called a “collectin” (collagen-lectin), which protects against viruses, fungi and bacteria [35].

It has been reported that lung damage may contribute to, or be coincident with, the cardiovascular effect induced by PM_2.5_ exposure. For this reason, we evaluated the MBP as a cardiovascular response to PM_2.5_ on the basis that it represents the peripheral blood pressure and blood perfusion of the organs. The MBP showed an augment after subchronic exposure to PM_2.5_. These changes were confirmed when we compared the measurements with the initial measurements of the PM_2.5_ and FA groups. As a consequence of an increased MBP, organ blood perfusion could be affected. The systemic blood pressure and kidneys have a close physiological relationship. Approximately 20% of the total renal blood flow supplies the glomeruli and 80% supplies the tubules [36]. According to these data, the peritubular capillaries could be affected and suffer damage due to the MBP increase.

In the kidneys, autonomic control of blood pressure is maintained as a constant perfusion flow along peritubular capillaries by the sympathetic nervous system (SNS), which innervates the three major renal neuro-effectors, the juxtaglomerular granular cells, the tubular epithelial cells, and the renal vasculature [7, 37]. It has been observed that the SNS is over-activated in hypertensive states and exposure to PM_2.5_ could stimulate its activity [38, 39]. Stimulation of the renal sympathetic nerve activity (RSNA) leads to an increase in renin secretion, and the increased renal tubular sodium reabsorption decreases the renal blood flow [40]. As an alternative hypothesis, PM_2.5_ exposure may impact the RSNA by affecting the circumventricular organs within the central nervous system because these brain structures lack a normal blood–brain barrier and are sensitive to circulating Ang-II [41]. In the context of the effect of PM on the RAS and KKS described in our previous report [6], systemic stimulation of the circumventricular organs by Ang-II may contributes to the stimulation of RSNA through its connections with sympathetic preganglionic neurons in the intermediolateral column of the spinal cord to the efferent sympathetic fibers that innervate the kidneys.

Eight weeks of exposure to PM_2.5_ or FA did not affect the body weights or kidney relative weights in the rat experimental model used in this study. However, exposure to PM_2.5_ modified water consumption as well as the urinary flow rate. The water consumption data suggested a possible dehydration of the animals in the PM_2.5_ group. The possible roles of airflow and the time in the hermetic chamber in these changes were excluded because both, the PM_2.5_ and FA groups, were exposed under the same experimental conditions of airflow (2.5 L min^−1^) and time (5 h). For this reason, it can be suggested that: 1) exposure to PM_2.5_ could induce a loss of water within the airways through the hydroscopic properties of the PM_2.5_ that stimulated thirst and water intake, and both phenomena could have contributed to the increase of urinary flow; 2) neurohormonal stimulation (angiotensin-aldosterone-vasopressin) could promote increased water consumption and/or increase urine production through the autonomous nervous system and the central nervous system (CNS). The last elements of this pathway to be affected are the circumventricular organs, which are sensitive to osmolality fluctuations and produce the sense of thirst [42]. A dehydrated state or a vulnerability to this condition has been observed in diseases such as cystic fibrosis, bronchitis and asthma. The pulmonary water lining is necessary for adequate pulmonary clearance and is involved in the hydration and protection of the pulmonary epithelium [43–45]. A dehydrated state resulting from exposure to PM_2.5_ may contribute to lung susceptibility to infection and diseases.

Although the urinary volume increased, the general urine parameters, such as pH and specific gravity, were not modified at the end of the subchronic exposure. However, surprisingly, the presence of hemoglobin in the urine from the PM_2.5_ group was observed. These data were consistent with the analysis of the urinary sediment, which showed the presence of erythrocytes was indicative of an isolated microscopic hematuria in the PM_2.5_-exposed group. This could have been the result of an effect on the renal microvascular circulation. However, there are many different causes of hematuria, including urinary infections, hypertension, and cancer, among other pathological states [46]. Further analysis must be performed to determine the origin of the red blood cells present in the urine and their association with particulate exposure.

The hydration state and the urinary sediment analysis suggest that the kidney function was compromised after exposure to PM_2.5_. The data for the plasma creatinine and eGFR levels support our hypothesis. Specifically, the plasma creatinine increased modestly and the eGFR decreased (approximately 15% compared to the FA group) after eight weeks of PM_2.5_ exposure. These results indicate that glomerular filtration could be compromised. Moreover, the early kidney biomarkers demonstrated a dysfunction in the reabsorption of proteins. Of the six proteins measured, the five non-albumin proteins were altered in the second week, which indicated that this particular week was a vulnerable stage for kidney physiological balance after PM_2.5_ exposure. Moreover, the most sensitive biomarkers were β2M and Cys-C, which showed differences beginning in the first week and continuing to the second, sixth and eight weeks. The presence of chronic renal disease is associated with the progression of CVD, and this relationship is exacerbated in terminally ill patients. However, changes in early kidney biomarkers, such as Cys-C in non-kidney diseases, have been associated with an increased rate of mortality from both CVD and non-CVD (pulmonary diseases, cancer, infections), as indicated by the quartiles of kidney function [47]. On the other hand, β2M, also used as an early kidney biomarker, has been reported to be independently associated with the mortality from heart attacks and strokes in patients with asymptomatic coronary atherosclerosis [48].

The use of serum creatinine and its depuration as an accurate measure of eGFR is unreliable. As reported by Endre and Westhuyzen (2008), for renal diseases such as acute kidney injury, serum creatinine is not a real-time marker during rapidly changing renal function and can vary widely with age, gender, and muscle metabolism and hydration status. Also, the tubular secretion of creatinine, which can contribute up to 50% as renal function declines, limits eGFR use as a kidney injury marker. The use of new molecular biomarkers increases the prognosis sensitivity of renal damage and also helps to specify the site where the damage occurs [49, 50]. The low association of renal damage and PM exposure could be related with the poor prognosis when creatinine parameters are used [18, 19]. New molecular kidney damage biomarkers used in the present study contribute to establish the nephrotoxic effect of PM_2.5_.

Previously, we reported that PM_2.5_ induced up-regulation of AT_1_R in lung and heart tissues. This receptor is involved in cardiovascular diseases, such as hypertension. The elements of the RAS are expressed constitutively and are over-expressed in the pathological states of kidney. As expected, we observed the up-regulation of the levels of the AT_1_R, and ACE proteins; however, discrepancies in the mRNA levels and proteins were observed. Protein and mRNA differences of AT_1_R and ACE levels were observed; the discrepancy could be explained by other molecular mechanism involved in transcription and translational cellular processes. Cis-acting sequences and glucocorticoid-responsiveness elements control AT_1_R gene mRNA translation, also, the AUG codon in the 5′-leader of AT_1_R transcript is involved in AT_1_R protein increment without the induction of mRNA [51].

Based on this background, the mRNA and proteins for AT_1_R and ACE, the RAS genes expressed in the kidneys, were modulated after a subchronic exposure to PM_2.5_, which suggested that PM_2.5_ was able to induce RAS up-regulation in organs, including the kidneys, that are distal to the deposition of the particulates.

Concerning the KKS, an endocrine system that counterbalances RAS, a decrease in the kallikrein released into the urine of hypertensive patients with primary aldosteronism has been previously reported [52], but in hypertensive rat models, a decrease in the expression of this enzyme has been observed [53, 54]. The KLK-1 decrease is associated with renal dysfunction, and the administration or the transfection of KLK-1 reverses the fibrosis and glomerular hypertrophy; reduces inflammatory cell infiltration, apoptosis, and TGF-β expression; and also decreases oxidative stress due to the inhibition of NADPH oxidase activity and the increase in the nitric oxide concentration. Thus, renal function recovery contributes to cardiovascular improvement. All of these effects were abolished by the administration of icatibant, a B_2_R inhibitor, indicating that the beneficial effects of KLK-1 are mediated by the generation of bradykinin and the B_2_R pathway [55, 56].

The measurements of the kallikrein mRNA and protein levels were down-regulated in the kidney tissue after PM_2.5_ exposure; this decrease could be explained by a release of the enzyme into the urine as previously reported [54]. On the other hand, the KLK-1 gene has been reported to contain an intron-III retention splice variant that has a high GC content sequence. This feature of intron-III might indicate that this region of the gene is more susceptible to transcription factor binding, resulting in higher transcriptional activity, which may depend on epigenetic regulation [57].

The role of bradykinin is to mediate vessel vasodilatation through the ligand-dependent activation of the bradykinin receptor type-2 (B_2_R); however, B_1_R can be induced by cytokines during inflammatory processes. PM_2.5_ exposure increased the B_1_R mRNA and protein levels in the kidney, which suggested that this organ may suffer from an inflammatory process and continuous oxidative stress on the basis that B_1_R stimulates the production of inducible nitric oxide enzyme [55–60].

In this context, to demonstrate oxidative stress involved in kidney damage after PM_2.5_ exposure, we analyzed the mRNA induction of genes associated with the antioxidant response and their protein levels. Our results showed an induction of *Nrf2* and a down-regulation *of Hmox1* and *Sod2*. The protein levels of all of the analyzed genes were decreased. To demonstrate that glutathione synthesis was also affected by PM_2.5_ exposure, we evaluated the catalytic subunit of the γ-GCL protein and observed a marginal induction. Our results show low levels of enzymatic antioxidants after the subchronic exposure, which could be due to: 1) the possible regulation of enzymatic antioxidants by secondary mediators, such TGF-β, which down-regulate catalase, glutathione reductase and SOD enzymes in the kidney [61]; 2) the susceptibility of antioxidant proteins to oxidation by oxygen and nitrogen radical molecules [62]; and 3) the loss of Nrf-2 activity due to severe kidney damage without de novo synthesis and the restoring of antioxidant levels [63].

One of the most important hypothesis of PM_2.5_ toxicity is the induction of inflammatory process in lung tissue with the release of cytokines, such as IL-6, TNFα, IL-8, amongst others [2, 3, 61, 62]. We evaluated kidney inflammation by measuring the cytokine levels in protein extracts of the kidney cortex, lower levels were observed in the PM_2.5_ exposed group, and in urine levels were lower than the assay detection limit, in contrast with PM-toxicity hypothesis. This results could be explained by the fact that cytokines are removed from the tubular lumen by endocytosis/metabolism along the proximal tubules [63]. Moreover, it has been reported that isolated proximal tubules have the ability to reduce the human recombinant IL-6 concentration under normoxic or hypoxic conditions [64].

In contrast to the other cytokines, the expression of RANTES increased. This effect probably occurred because this chemokine has the longest half-life among the cytokines and chemokines evaluated or because the induction of RANTES occurred in a more delayed manner. In human mesangial cell lines under a pro-inflammatory condition obtained by stimulation with TNFα or a combination of TNFα and IL-1β, a slow mRNA expression of RANTES after 12 h of stimulation and increased protein expression after 36 h was observed [65, 66].

The renal cytokine levels of the PM_2.5_ group were lower than in the FA group. The expression of chemokine genes can be inhibited by glucocorticoids as well as by cytokines, such as TGF-β and prostaglandins [67]. We observed an increase in the TGF-β protein levels in the kidney cortex extracts. This observation could explain part of the effect on the cytokines as well as on the expression of the antioxidant enzymes.

The present evidence indicates that inhalation of PM_2.5_ is able to induce physiological and molecular responses in the kidney. We were also interested to determine whether exposure to PM_2.5_ could cause tissue lesions. Through the histological study using H&E staining, we observed intertubular infiltration and a reduction in the height of the tubular simple cuboidal epithelium. The significant changes observed in tubular height suggest tubular damage, this type of tubular lesions has been described in human acute kidney injury [68]. This last observation was supported by the evaluation of KIM-1, a specific biomarker of proximal tubule damage that is undetectable in healthy kidneys but is induced during renal injury [69]. The analysis showed a 4-fold increase in KIM-1 in the PM_2.5_ group compared to the FA group. Thus, exposure to PM_2.5_ induced injury to the proximal tubule epithelium.

Our data indicated that during the eight weeks of exposure to PM_2.5_, the kidneys undergo constant damage, but it is probable that a response to repair the damage is equally triggered. As mentioned earlier, an increase in TGFβ in the PM_2.5_ group was observed at the end of exposure. TGFβ is known to be involved in tissue repair after injury in which it promotes the deposit of components of the extracellular matrix, such as collagen. However, continuous uncontrolled TGFβ production can increase the extracellular matrix and generate fibrotic tissue [70]. We observed a probable repair process in the kidneys, although the *Col3a1* levels decreased, Masson’s Trichromic staining showed an increment in the collagen deposit area on renal tissue. This evidence supports the idea that the subchronic exposure to PM_2.5_ induces kidney injury, and consequently promotes a repair process, by the presence of TGF-β and the deposit of collagen, however, the persistent exposure to PM_2.5_ could promote a pro-fibrotic state and a kidney dysfunction.

Changes in urine biomarkers fluctuate along the eight-week exposure period, particle concentration in the exposure chambers do not correlate with the urinary early kidney damage biomarkers. Despite having enriched the PM_2.5_ concentration between nine—and twenty-fold, we did not observe correlation of the highest PM_2.5_ concentration corresponding to the greatest increase in early kidney damage biomarkers. However, the changes or fluctuation in the intrinsic redox activity of PM_2.5_ and the endotoxin content did show a statistical correlation (Additional file 1: Table S2). Endotoxin data correlated with a 60% (plus/minus) of the urinary kidney damage biomarkers (AGP, β2M, Cys-C, EGF and NGAL) evaluated and the redox activity to a 30% (plus/minus) with AGP and Cys-C. Thus, PM mass does not entirely explain the observed kidney effects, PM components and their biological and chemical stimulation are relevant factors in PM toxicity. Analysis of the endotoxin content and the redox activity (DTT assay) of PM_2.5_, as general parameters of particulate reactivity, to better understand the relation of PM_2.5_ concentration exposure and biomarker results was performed. It has previously been reported that acute intraperitoneal or intravenous administration of endotoxin (lipopolysaccharide) in rodent models can induce acute renal failure [71, 72] through stimulation of toll-like receptor-type 4 and tumor necrosis factor. Thus, it is possible that in our model the PM_2.5_ endotoxin content, after the deposit and accumulation of PM_2.5_ in lung, contributed to the deleterious effect in the kidneys through circulation. Endotoxin exposure can induce an inflammatory response as a consequence of an acute exposure and contribute to the development of tolerance after repeated doses [73]. Nevertheless, kidney damage biomarkers showed a significant correlation with PM_2.5_ endotoxin content indicating kidney’s sensitivity to endotoxin and suggesting that kidney injury could, in part, be explained by it. However, we do not discard that other components of PM, inorganic or organic, play a role as enhancers or coadjuvants in kidney-induced damage, also the time of exposure can influence this kidney response, and other factors intrinsic of particulates (such source and composition) and of population (age and gender). Moreover, based on our data, acute PM_2.5_ exposure could be the starting point of the kidney injury but would not compromise kidney function. However, repeated and longer term exposure induces kidney injury exceeding the capability of organ defense against pathological conditions, that in a condition of pre-existing diseases, related with cardiovascular or renal systems, could accelerate the renal dysfunction.

In summary, exposure to PM_2.5_ induced its pulmonary toxic effect mainly through oxidative stress and inflammatory processes due to the interaction between the particles and lung tissue. This initial response can trigger the release of secondary mediators (cytokines and oxygen free radicals) that may modulate the expression of two of the major endocrine systems involved in the regulation of vascular tone, the RAS and KKS. This modulation produces a second level of damage in which the impaired balance of these endocrine systems promotes the over-expression of AT_1_R [6]. We have demonstrated kidney damage through the analysis of early kidney biomarkers (KIM-1, Cys-C, β2M), histological changes (tubular height, collagen deposit) and the induction of RAS/KKS endocrine genes (AT_1_R, B_1_R), and blood pressure increment, after the subchronic exposure to PM_2.5_. Under the reasoning that exposure to PM_2.5_ triggers lung inflammation that can be translocated to circulation and implicates other organs, we tested cytokine release in serum and kidney tissue. Cytokine levels in serum were under the test detection limit, and cytokines in kidney tissue were below control group levels. Further analysis to demonstrate if the release of secondary mediators from the lung into blood circulation are involved in kidney damage are needed; also temporality of the immunologic modulation of cytokines should be considered. Furthermore, kidney damage is a consequence that could be triggered beyond the inflammatory response and other non-immunological mechanisms could be implicated such as: oxidative stress, endocrine disruption, peritubular microvascular damage from blood pressure changes, and ANS misbalance.

Finally, we propose that the kidney can subsequently exacerbate the initial effects. Several hypotheses can be established to determine how the effects on the kidneys contribute to the biological effects of PM_2.5_. These include modulation of the autonomic nervous control, the ionic balance and the hydration state, among other effects. Renal alterations induced by exposure to PM_2.5_ might contribute to the cardiopulmonary PM_2.5_ toxicity through feedback of the initial effect in several ways. The strongest evidence for this hypothesis is that patients with acute kidney injury have an increased incidence of respiratory failure; the details of this pathological process are not completely understood. However, in acute ischemic kidney injury or bilateral nephrectomy in IL-6 knockout mice, it has been established that neither surgical procedure induces a pulmonary effect compared to the wild-type mice [74, 75]. Thus, damage to the lungs and heart generated by PM_2.5_ could contribute to renal dysfunction.

## Conclusions

The repeated exposure to PM_2.5_ not only induced biochemical and physiological responses associated with cardiopulmonary toxicity but also induced early kidney dysfunction, which was characterized by a reduced level of eGFR, presence of hematuria, increased urinary early kidney damage biomarkers, imbalance in the RAS/KKS response, the impairment of the antioxidant response with a reduction of enzymatic antioxidants, suppression of cytokine expression and finally, histological lesions with the presence of intertubular cell infiltration and the presence of collagen, which indicated a pro-fibrotic damage. The early kidney damage could be associated with the intrinsic PM_2.5_ oxidant reactivity and the endotoxin content of PM_2.5_. The present study demonstrates the effects of subchronic PM_2.5_ exposure on the physiology of the kidney. Furthermore, the data presented here suggests that the kidneys are a novel target organ that is involved in cardiopulmonary responses and could aggravate systemic effects. This process requires further study.

## Additional file

Additional file 1: Table S1. Blood pressure, kidney and urine parameters after acute exposure to PM_2.5_. **Table S2.** Pearson correlation of particulate endotoxin content and intrinsic oxidative particulate activity (DTT assay) with early kidney damage biomarkers. (DOCX 15 kb)

## Abbreviations

Ace: Angiotensin converting enzyme mRNA
ACE: Angiotensin converting enzyme
AGP: α-1-glycoprotein
AT_1_R: Angiotensin receptor type-1
At1r: Angiotensin receptor type-1 mRNA
B_1_R: Bradykinin receptor type-1
B_1_r: Bradykinin receptor type-1 mRNA
B_2_R: Bradykinin receptor type-2
BALF: Bronchoalveolar lavage fluid
Col3a1: Pro-collagen-III mRNA
CVD: Cardiovascular diseases
Cys-C: Cystatin-C
DTT: Dithiothreitol
EGF: Epidermal growth factor
eGFR: Estimated glomerular filtration rate
FA: Filtered air
GUE: General urine exam
Hmox1: Heme oxygenase type-1 mRNA
HO-1: Heme oxygenase type-1
KIM-1: Kidney-injury-molecule type-1
KKS: Kalikrein kinin system
KLK-1: Kallikrein-1
Klk-1: Kallikrein-1 mRNA
MBP: Mean blood pressure
MΦ: Macrophage
NGAL: Neutrophil gelatinase-associated lipocain
Nrf-2: Nuclear factor (erythroid-derived 2)-like 2
Nrf2: Nuclear factor (erythroid-derived 2)-like 2 mRNA
PM_2.5_: Particulate matter of less than 2.5 μm
RAS: Renin angiotensin system
SOD-2: Superoxide dismutase type-2
Sod2: Superoxide dismutase type-2 mRNA
SPA: Surfactant protein-A
TGF-β: Transforming growth factor-beta
β2M: β-2-microglobulin
γ-GCSc: Heavy chain of gamma-glutamyl cysteine ligase
