# Supplementary material for: Early kidney damage induced by subchronic exposure to PM2.5 in rats
Source: Part Fibre Toxicol. 2016 Dec 12;13:68. doi: 10.1186/s12989-016-0179-8 (PMC5154051; doi:10.1186/s12989-016-0179-8)
Supplement: Additional file 1: Table S1. — Blood pressure, kidney and urine parameters after acute exposure to PM2.5. Table S2. Pearson correlation of particulate endotoxin content and intrinsic oxidative particulate activity (DTT assay) with early kidney damage biomarkers. (DOCX 15 kb) [file 12989_2016_179_MOESM1_ESM.docx]

**Supplementary Material**

| **Table 1. Blood pressure, kidney and urine parameters after acute exposure to PM_2.5_.** | | | |
| --- | --- | --- | --- |
| Parameter | FA | PM_2.5_ | P-value |
| Median artery blood pressure (mmHg) | 102.5  (101.3 – 114.5) | 125  (120.5 – 136.5) | 0.003 |
| Urinary flow  (µl/min/100 g weight) | 3.2  (3.2 – 7) | 3.9  (2.4 – 10.4) | 0.4 |
| Kidney relative weight | 0.68  (0.64 – 0.73) | 0.69  (0.67 – 0.75) | 0.32 |
| Plasma creatinine  (mg/ml) | 0.7  (0.5 – 0.98) | 0.7  (0.7 – 0.9) | 0.44 |
| pH | 7.0  (6.4 – 7.6) | 6.8  (6.5 – 7.1) | 0.3 |
| Urine specific gravity | 1.013  (1.01 – 1.015) | 1.015  (1.01 – 1.02) | 0.13 |
| Hematuria | 0:6 | 1:6 | 0.34 |
| The data are shown as the median followed by the 25-75 quartile interval in parentheses. | | | |

| **Table 2. Pearson correlation of particulate endotoxin content and intrinsic oxidative particulate activity (DTT assay) with early kidney damage biomarkers** | |
| --- | --- |
| **Correlation** | Correlation results |
| **DTT vs Endotoxin** | 0.6145  (-0.08089 – 0.9081)  0.0391 |
| **Endotoxin vs Albumin** | 0.2401  (-0.1316 – 0.5526)  0.1006 |
| **Endotoxin vs AGP** | 0.6566  (0.3881 – 0.8224)  < 0,0001 |
| **Endotoxin vs β2M** | 0.6271  (0.3446 – 0.8054)  <0.0001 |
| **Endotoxin vs Cys-C** | 0.6938  (0.4446 – 0.8433)  < 0.0001 |
| **Endotoxin vs EGF** | 0.7588  (0.5483 – 0.8788)  < 0.0001 |
| **Endotoxin vs NGAL** | 0.5582  (0.2476 – 0.7647)  0.0007 |
| **DTT vs Albumin** | 0.06082  (-0.3062 – 0.4121)  0.3748 |
| **DTT vs AGP** | 0.3497  (-0.01218 – 0.6306)  0.0291 |
| **DTT vs β2M** | 0,2991  (-0.06867 – 0.5953)  0.0542 |
| **DTT vs Cys-C** | 0.3581  (-0.002634 – 0.6363)  0.026 |
| **DTT vs EGF** | 0.3218  (-0.04364 – 0.6112)  0.0415 |
| **DTT vs NGAL** | 0.2711  (-0.09888 – 0.5753)  0.0736 |
| **Data are show as: Pearson R; 95% of confidence interval in brackets and Statistical significance *p<0.05*.** | |
